# Editorial: Physico-Chemical Control of Cell Function

**DOI:** 10.3389/fphys.2019.00355

**Published:** 2019-04-03

**Authors:** Alberto Rainer, Giancarlo Forte, Cesare Gargioli

**Affiliations:** ^1^Department of Engineering, Università Campus Bio-Medico di Roma, Rome, Italy; ^2^Center for Translational Medicine, International Clinical Research Center, St Anne's University Hospital, Brno, Czechia; ^3^Department of Biology, Tor Vergata University, Rome, Italy

**Keywords:** ECM, extra cellular matrix, physico-chemical parameter, cell biology, scaffold matrix, mechanical and topographical cues

The mechanical properties of the extracellular matrix (ECM) are fundamental in controlling cell behavior. Evidence has been given that ECM mechanics and nanotopography are at least as important as the biochemical cues for cells to decide about their survival, proliferation, migration, and fate (Engler et al., 2006).

The interplay between cells and the ECM is continuously controlled at the cell level in a dynamic way and is tightly controlled by negative and positive feedback loops.

Cells synthesize the raw components of the ECM and remodel it; in turn, ECM impacts on cell function by providing chemical, topographical and mechanical hints (Calvo et al., 2013). This consideration calls for an increasing attention toward the investigation of new and performing biomimetic scaffold matrices mirroring ECM mechano-physical and chemical properties (Fuoco et al., 2016).

Here we propose a collection of original articles, reviews and technical reports having the aim to provide readers with a comprehensive overview on: (1) the role and nature of mechanical cues arising from ECM in determining cell function; (2) the molecular mechanisms underlining cell response to ECM mechanics and nanotopography; (3) the current experimental tools used to mimic ECM dynamics as well as to observe and interpret cell mechanosensing. Also, we offer few significant contributions regarding the generation on new *in vitro* and *in silico* models of cell-matrix interaction.

In a comprehensive review, Martino et al. revise the current knowledge on intracellular mechanosensing pathways using a concentric approach starting from the site of cell-ECM physical interaction, the focal adhesion, and going down to the nucleus, where specific genetic programs are activated in response to mechanical stimuli. The response to mechanical challenge has been recently shown to entail the reinforcement of cell-matrix interaction and the development of cell force (Nardone et al., 2017).

In this context, the use of atomic force microscopy (AFM) allows for nanoscale mapping of cell mechanical properties and topography in living cells, thus allowing for subcellular and live monitoring of cellular processes, like cytoskeleton rearrangement and cell shaping.

Caluori et al. question the benefits and limitations of currently used mechanical models for post-processing and interpretation of AFM data in order to quantify the impact of such models on the final evaluation of cellular elasticity.

The versatility of AFM platform allowed Golan et al. to propose nanoindentation as a strategy to monitor and characterize frozen cell recovery by measuring changes in their elastic properties.

Mechanobiology-on-a-chip is regarded as the future avenue to set up *in vitro* physiological and pathological models of *in vivo* conditions. In this Topic, Ergir et al. critically revise the recent advances in lab-on-a-chip platforms in the context of mechanobiology models and elaborate on the next-generation physiological and pathological organ-on-a-chip models.

Additionally, Basoli et al. introduce and critically discuss the recent advances in optical, magnetic, and acoustic tweezers to investigate the mechanobiology of living cells. Together with active and sensing substrates produced by manipulating biomaterials chemistry or by microfabrication techniques, strategies based on such technologies appear to expand the potential of classical AFM approaches.

Among the tools used to study cell-ECM interaction, artificial, and natural scaffolds play an important part. A major issue in 3D scaffold preparation is the diffusion of nutrients and oxygen to the core of the construct. This limitation poses serious concerns when parenchymatic substitutes are planned.

To overcome this problem, Zirath et al. developed microfluidic devices containing embedded sensor arrays and able to monitor local oxygen levels. The devices are proposed to investigate oxygen consumption rates of hydrogel-based cell cultures, the establishment of oxygen gradients within cell culture chambers and the influence of microfluidic material, surface coatings, cell densities, and medium flow rate on the respiratory activities of selected cell types.

In a classical mechanobiology study, Rufaihah et al. elaborate on the effects of scaffold stiffness on fetal mesenchymal stem cells by using semi-synthetic hydrogels made of PEG-Fibrinogen. On the same line, Williams et al. propose an original study in which fiber-reinforced composite hydrogels were fabricated by far field electrospinning with the aid of guiding electrodes and gravity-assisted, droplet-based system to deposit the hydrogel component. The authors show that the fibrous component is able to slightly increase scaffold elastic modulus, thus controlling cell morphology.

In this context, the original paper by Rüger et al. addresses the suitability of 3D explant culture in fibrin hydrogels as a disease-related platform to integrate complex cell-cell and cell-ECM interactions with associated paracrine signaling patterns.

One of the most represented components in ECM is collagen, the molecule responsible for the scaffolding properties of the matrix and whose derangement has been associated to a number of severe pathological conditions.

By investigating the *in vitro* behavior of fibroblasts obtained from a Col6a1^−/−^ mouse, the group of Castagnaro et al. emphasizes the critical effects of collagen VI on cell autophagy regulation and survival, thus offering a model for pathologies based on its depletion.

The body district being more affected by collagen VI depletion is muscle. We host in the Topic three papers related to muscle biology: the group coordinated by Maleiner et al. provides a clear and comprehensive review of skeletal muscle development, while emphasizing the need for novel, more representative models of muscle diseases *in vitro*.

Additionally, the original paper by the group of Thorrez et al. describes an original protocol based on fibrin and collagen I scaffolding materials to control the visco-elastic properties of adult skeletal muscle progenitor-derived bioartificial muscles. The fine-tuning of the components provided clues to find a compromise between cell differentiation and the angiogenesis required to support this process.

The milieu cells are exposed to also includes surrounding cells and the products they release to exert paracrine effects.

Extracellular vesicles (EVs), a broad category including exosomes, microvesicles, and apoptotic bodies, have been lately subject to intense investigation as mediators of cell-to-cell paracrine communication at short and long distances.

Rackov et al.'s group revises the recent discoveries that EVs can transport and release enzymes involved in ECM remodeling, thus actively contributing to cell function and tumor spreading.

Tumor microenvironment is characterized by increased hyaluronan and altered integrin binding, this condition being credited of favoring metastatization. While Zapp et al. report on a new method to prepare self-assembled monolayers on gold surfaces, co-presenting the cell adhesive RGD motif and small hyaluronan molecules, to investigate integrin binding, Sun et al.'s group adopted a fully *in silico* bioinformatics approach to identify the enriched and dysregulated pathways in 362 hepatocellular carcinoma tissues, which, as expected, include ECM-responsive elements.

Vitiello et al. propose an original study in which the repurposing of safinamide, a monoamine oxidase inhibitor already in the clinical pipeline for neurological diseases, is used to treat dystrophic patients. They demonstrate the efficacy of the drug *in vitro* by testing the compounds on myogenic culture derived from *mdx* dystrophic mice and Duchenne Muscular Dystrophy (DMD) patients.

While this paper takes advantage of DMD patient-derived myoblasts, Spitalieri et al. describe the generation of the first two induced pluripotent stem cell lines coming from patients diagnosed with myotonic dystrophy type 2 and successfully managed to push them to neuronal differentiation, thus producing a new, valuable patient-specific *in vitro* disease model. This disease model complements the study by the group of Dobrowolny et al., investigating the role of mutations in superoxide dismutase 1 contribution to amyotrophic lateral sclerosis phenotype.

Finally, underlining the need for new *in silico* predictive tools, the paper proposed by Merino-Casallo et al. adopts a Bayesian optimization to predict the behavior of cells in a 3D environment by mimicking the existence of controlled mechano-chemical constraints.

In conclusion, this topic collects a number of interesting studies dealing with the manifold aspects of cell-matrix interplay, showing knotty lapels regarding the intimate interaction tying ECM and cells. This interaction is yet to be fully untangled.

## Author Contributions

All authors listed have made a substantial, direct and intellectual contribution to the work, and approved it for publication.

### Conflict of Interest Statement

The authors declare that the research was conducted in the absence of any commercial or financial relationships that could be construed as a potential conflict of interest.

## References

[B1] Calvo F. Ege N. Grande-Garcia A. Hooper S. Jenkins R. P. Chaudhry S. I.. (2013). Mechanotransduction and YAP-dependent matrix remodelling is required for the generation and maintenance of cancer-associated fibroblasts. Nat. Cell Biol. 15, 637–646. 10.1038/ncb2756 23708000 PMC3836234

[B2] Engler A. J. Sen S. Sweeney H. L. Discher D. E. (2006). Matrix elasticity directs stem cell lineage specification. Cell 126, 677–689. 10.1016/j.cell.2006.06.044 16923388

[B3] Fuoco C. Cannata S. Gargioli C. (2016). Could a functional artificial skeletal muscle be useful in muscle wasting? Curr. Opin. Clin. Nutr. Metab. Care 19, 182–187. 10.1097/MCO.0000000000000271 26910194

[B4] Nardone G. Oliver-De La Cruz J. Vrbsky J. Martini C. Pribyl J. Skládal P.. (2017). YAP regulates cell mechanics by controlling focal adhesion assembly. Nat. Commun. 8:15321. 10.1038/ncomms15321 28504269 PMC5440673

